# The Effect of Aerobic Exercise on Neuroplasticity within the Motor Cortex following Stroke

**DOI:** 10.1371/journal.pone.0152377

**Published:** 2016-03-28

**Authors:** Kate Murdoch, Jonathan D. Buckley, Michelle N. McDonnell

**Affiliations:** 1 International Centre for Allied Health Evidence, Sansom Institute for Health Research, School of Health Sciences, University of South Australia, Adelaide, South Australia, Australia; 2 Alliance for Research in Exercise, Nutrition and Activity (ARENA), Sansom Institute for Health Research, University of South Australia, Adelaide, South Australia, Australia; University of Ottawa, CANADA

## Abstract

**Background:**

Aerobic exercise is associated with enhanced plasticity in the motor cortex of healthy individuals, but the effect of aerobic exercise on neuroplasticity following a stroke is unknown.

**Objective:**

The aim of this study was to compare corticomotoneuronal excitability and neuroplasticity in the upper limb cortical representation following a single session of low intensity lower limb cycling, or a rest control condition.

**Methods:**

We recruited chronic stroke survivors to take part in three experimental conditions in a randomised, cross-over design. Corticomotoneuronal excitability was examined using transcranial magnetic stimulation to elicit motor evoked potentials in the affected first dorsal interosseus muscle. Following baseline measures, participants either cycled on a stationary bike at a low exercise intensity for 30 minutes, or remained resting in a seated position for 30 minutes. Neuroplasticity within the motor cortex was then examined using an intermittent theta burst stimulation (iTBS) paradigm. During the third experimental condition, participants cycled for the 30 minutes but did not receive any iTBS.

**Results:**

Twelve participants completed the study. We found no significant effect of aerobic exercise on corticomotoneuronal excitability when compared to the no exercise condition (*P* > 0.05 for all group and time comparisons). The use of iTBS did not induce a neuroplastic-like response in the motor cortex with or without the addition of aerobic exercise.

**Conclusions:**

Our results suggest that following a stroke, the brain may be less responsive to non-invasive brain stimulation paradigms that aim to induce short-term reorganisation, and aerobic exercise was unable to induce or improve this response.

## Introduction

Stroke is leading cause of adult disability in Australia [1] and the US [2]. It is estimated that 7 million American adults are living with stroke which cost the US healthcare system $33.6 million in 2011 [2]. In Australia, the majority (88%) of stroke survivors live in the general community [2]. However, of these survivors, 64% need assistance with health care, 58% with mobility and 47% with self-care [2]. This is a significant burden for individuals, families and carers, and meeting these care needs is going to be a substantial problem in the future as the population ages [3, 4].

Exercise participation, in particular aerobic exercise, has long been known to have a wide range of physical and mental benefits. These include reducing the risk of cardiovascular disease, some cancers, obesity, anxiety and depression [5]. Regular physical activity and exercise are also recommended in national guidelines to improve physical function and as part of risk factor management for secondary prevention of stroke [6, 7]. More recently however, evidence has emerged relating to benefits of exercise for cognitive function in healthy adults, with studies demonstrating enhanced cognitive flexibility [8], improved learning [9], improved executive control and improved executive function [10, 11] as well as the potential to improve motor learning [12, 13]. Evidence is emerging that exercise can also improve cognition in stroke survivors [14–16]. These cognitive benefits may be, at least in part, a result of exercise promoting neuroplasticity in the hippocampus [17–19], a key structure involved in learning and memory.

Evidence in healthy older adults suggests that higher cardiorespiratory fitness levels are associated with greater grey matter volume in the hippocampus, with several longitudinal studies confirming that physical activity can increase grey matter volume [20]. In addition to this evidence of exercise induced neuroplasticity in the hippocampus, there is growing evidence that aerobic exercise can also promote neuroplastic changes in the motor cortex [19], and in particular low intensity aerobic exercise [18]. Due to the vital role of neuroplasticity in regaining function and skill for people following a stroke [21], an intervention which promotes motor cortical reorganisation may have potential to improve functional outcomes, and quality of life, in stroke survivors.

The non-invasive brain stimulation technique transcranial magnetic stimulation (TMS) is one approach to evaluate neuroplasticity following stroke. TMS excites the fast conducting corticomotoneuronal pathway and can be used to measure changes in effectiveness of synaptic connections, or short-term plasticity in the brain [22]. There are several forms of patterned TMS which can facilitate long-term potentiation-like (LTP-like) changes at the cortical level. Intermittent theta-burst stimulation (iTBS, described below) when applied in a single session can result in a transient increase in motor cortical excitability [23]. It has been suggested that repeated application of iTBS and other non-invasive brain stimulation techniques to the damaged motor cortex could enhance the response to rehabilitation following stroke [24], which was supported by one randomised controlled trial [25], but not another [26].

The primary aim of this study was to evaluate the effects of a single bout of low intensity aerobic exercise on neuroplasticity in the motor cortex of people who had suffered a stroke at least six months prior. Secondary aims were to investigate whether exercise alone influenced corticomotoneuronal excitability in stroke survivors, and whether iTBS alone influenced neuroplasticity following stroke.

## Methods

### Participants

We recruited participants aged 18–80 years old who experienced a stroke at least six months prior to participation. Participants were recruited from the community via flyers circulated to exercise and stroke support groups. They were excluded if they had any cardiac or other medical problems that would prevent them from performing 30 minutes of low-intensity cycling exercise (for example unstable angina, uncontrolled hypertension, atrial fibrillation, heart failure), any conditions that were contraindicated for TMS, or no muscle response to TMS (see below). Participants were also excluded if they were taking beta-blocker medication as this would have interfered with the ability to determine a suitable exercise intensity based on ratings of perceived exertion (RPE) [27]. All participants were reviewed by their local medical practitioner prior to participation in the study and provided written, informed consent. This study was approved by the local ethics committees and conformed to the Declaration of Helsinki.

### Study design

**Fig 1:**
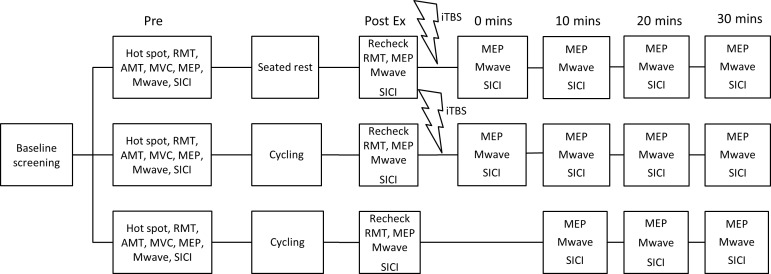
Timeline of interventions. Timeline indicates order of conditions and measures that were collected, with all participants taking part in all three conditions. Measures were taken before (T_pre_) and post exercise (T_post_), and at 0 (T_0_), 10 (T_10_), 20 (T_20_) and 30 mins (T_30_) following intermittent theta burst stimulation (iTBS). Note during the low intensity cycling condition no iTBS was delivered, therefore measures of cortical excitability were completed at 0 (T_post_), 10 (T_10_), 20 (T_20_) and 30 mins (T_30_) post exercise intervention only. [RMT = Resting motor threshold; AMT = Active motor threshold; MVC = Maximal voluntary contraction; MEP = Motor evoked potential; Mwave = Maximal motor wave; SICI = short interval intracortical inhibition]

A within-subject, repeated-measures, randomised crossover design was used with three intervention conditions. Each condition was separated by at least one week to prevent potential carry-over effects on corticomotoneuronal excitability [28] and a random number generator was used to determine the order of testing sessions. All testing occurred in the afternoon to account for diurnal variations in cortisol [29]. At each test session, baseline measures of cortical excitability were recorded using TMS. Following this, measures were taken immediately post exercise/rest ± intermittent theta burst stimulation (iTBS, described below), and every 10 minutes for a total of 30 minutes. In conditions that involved use of iTBS, measures were taken post exercise/rest condition, and pre application of iTBS. See Fig 1 for timeline of experiments.

### Baseline Assessments/Screening

The Short Form International Physical Activity Questionnaire (IPAQ) [30] was used to screen participants’ baseline physical activity levels because of the known enhanced motor cortical neuroplasticity for highly active individuals [19]. The IPAQ has been validated as a marker of fitness [31], and provides a reliable measure of physical activity in a variety of age groups and settings [32], including post-stroke [33, 34]. Although participants were not excluded if they participated in high levels of physical activity, baseline physical activity was considered in the analyses.

Two additional screening measures verified whether participants were suitable to undergo TMS. First, the TMS Adult Safety Screen was completed to identify participants who should be excluded due to reasons such as metallic implants in the head, epilepsy etc [35]. During the first test session, participants were further screened for eligibility to undergo the full TMS protocol. If TMS did not elicit a muscle evoked potential (MEP) of at least 0.5mV, at 80% of maximum stimulator output, participants were excluded.

### TMS

Electromyography (EMG) was used to objectively measure the muscle response to cortical stimulation (motor evoked potential or MEP), and therefore the excitability of the corticomotoneuronal projection to the hand. Participants were seated with the arm contralateral to the side of the brain affected by stroke resting in a pronated position on their lap. Skin was cleaned and prepared, and adhesive electrodes (Ag/AgCl) were placed over the first dorsal interosseous (FDI) muscle belly and the metacarpophalangeal joint in a belly-tendon montage to allow for surface EMG recordings. The FDI muscle was chosen as it is small, localised and easily identifiable using palpation, as well as having the ability to elicit recordable responses from TMS at lower stimulation intensities [36]. As this muscle is not involved in the lower limb cycling exercise, it is unlikely to be influenced by fatigue which can depress corticomotoneuronal excitability [37]. Further, it has been established that changes in corticomotoneuronal excitability, neuroplasticity and motor learning following exercise are evident in muscles not directly involved in the exercise [12, 18, 19, 27, 38]. EMG signals were amplified (1000x), bandpass filtered (20-1000Hz), digitized at a sampling rate of 2000Hz using a CED1401 interface (Cambridge Electronic Design, Cambridge, UK), recorded and stored on a computer for offline analysis using Signal 4.09 software (Cambridge Electronic Design, Cambridge, UK).

TMS pulses were applied through a figure-of-eight coil (outer wing diameter 90 mm) connected to a Magstim 200 magnetic stimulator (Magstim, Whitland, UK). The coil was positioned tangentially to the skull with the handle orientated posteriorly and at a 45-degree angle to the midsagittal line over the motor cortex. The optimal scalp site (on the stroke affected side of the brain) to evoke a response in the relaxed FDI was located and marked using a soft-tipped pen and determined for each session. The coil was held by hand throughout the experiment, and placement of the coil was regularly checked.

Motor threshold was determined according to the recommendations of the International Federation for Clinical Neurophysiology [39]. The resting motor threshold (RMT) was defined as the lowest stimulation intensity evoking MEPs of 0.05 mV in the relaxed FDI muscle in greater than 5 out of 10 consecutive stimulations. The active motor threshold (AMT) was determined using an air-cooled coil connected to a Magstim Super Rapid (Magstim, Whitland, UK) to deliver intermittent theta burst stimulation (iTBS) and the optimal scalp site when using this coil was determined separately and marked on the scalp. AMT was determined with the participant maintaining an isometric contraction of the FDI at approximately 20% of their maximum voluntary contraction (MVC). The MVC of the FDI was determined using a force transducer, with a participant producing a maximal precision grip force between the index finger and thumb for 3 seconds. Three maximal contractions were recorded, separated by a rest period of at least 30 seconds, with the largest of these recorded as the participant’s MVC. The AMT was defined as the lowest stimulation intensity to elicit MEPs of 0.2 mV in greater than 5 out of 10 consecutive stimulations. Participants were encouraged to view an oscilloscope during testing to help maintain the appropriate force for determination of active threshold.

Changes in motor cortical excitability were measured indirectly using single pulse TMS. This was performed as a baseline measure, post exercise/rest intervention, post iTBS (when delivered), and at 0 mins, 10 mins, 20 mins and 30 mins at 120% RMT to assess any stimulation-induced long-term potentiation-like (LTP-like) neuroplasticity of the motor cortex after iTBS. At each time point, fifteen MEPs were delivered at a rate of ~0.2 Hz and recorded.

Intermittent TBS (iTBS) was delivered as soon as practicable after exercise or rest [23]. The stimulation paradigm involved three low intensity high frequency pulses (50 Hz) applied every 200 ms for two seconds, then repeated every 10 seconds for a total of 190 seconds (i.e. 600 pulses) [23]. This paradigm utilises a low stimulation intensity (80% AMT) which is more suitable for stroke participants [40]. The iTBS was delivered using a Magstim Super Rapid magnetic stimulator (Magstim, Whitland, UK), which was applied to the optimal scalp site for motor cortex stimulation.

M-waves were recorded to enable normalisation of MEP amplitudes to changes in M-wave amplitude. The ulnar nerve was stimulated just proximal to the wrist, generating maximal compound muscle potentials within FDI. The optimal stimulation site was located using a bar electrode, before adhesive electrodes were applied to this site, and secured in position with tape for the remainder of the experiment. Stimuli were applied using a constant-current stimulator (DS7AH, Digitimer, Welwyn Garden City, UK). Each stimulus was a square wave pulse of 100 μs duration. The intensity of the stimulating pulse was increased until the M-wave amplitude reached a plateau. The intensity was then increased by a further 20% to ensure maximal M-wave amplitude, and maintained at this level for the remainder of the experiment. As previous exercise studies have demonstrated a decrease in maximal M-wave following exercise, possibly due to an increase in body temperature [41]. M-waves were recorded at each time point to maintain normalised MEP amplitudes. A total of five resting M-waves were recorded at each time point, and peak-to-peak amplitudes were calculated and averaged.

Paired pulse TMS at short inter-stimulus intervals in the form of Short Interval Cortical Inhibition (SICI) was used to investigate inhibitory circuits. A suprathreshold test pulse was preceded by a subthreshold conditioning pulse. This has previously been shown to reduce the amplitude of the test MEP due to the activation of gamma-amino butyric acid (GABA)_A_-mediated inhibitory interneurons in primary motor cortex [42, 43]. To evaluate these intracortical circuits, two inter-stimulus intervals were tested, 2 and 3 milliseconds (SICI2 and SICI3 respectively). The subthreshold conditioning pulse was set at 5% stimulator output below AMT and the suprathreshold test pulse at 120% RMT [44]. For SICI measurements, stimuli were applied to the same optimal skull site, using a figure-of-eight coil connected to a Magstim Bistim 2 programmable interval paired pulse stimulator (Magstim Co., Whitland, UK). This was assessed prior to the intervention to obtain baseline intracortical inhibition, and then at all time points throughout the experiment. At each time point 30 MEPs were recorded, with an average of 10 stimuli per state. SICI was quantified by obtaining the MEP peak-to-peak amplitude from each individual trial, averaging the MEPs from each state, then expressing the average conditioned response as a percentage of the unconditioned response.

### Exercise intervention

Exercise was performed on a recumbent cycle ergometer (Activate Series Recumbent Lifecycle® Exercise Bike, LifeFitness Australia, Mulgrave, Australia). Participants cycled at a cadence of 50 revolutions per minute (RPM) for 30 minutes and the resistance was graded to allow participants to maintain an RPE of 11–13 (light-somewhat hard) on the Borg 6–20 point scale [45]. This intensity was chosen to be a low-moderate intensity exercise, within the recently-updated recommendations for stroke survivors [6]. Participants were asked to maintain a relaxed posture in the hand being assessed for MEPs during exercise. Heart rate and RPE were recorded every five minutes throughout exercise. The control condition consisted of participants remaining resting in a seated position for 30 minutes.

### Data analysis

Data analysis was completed offline using Signal software. Trials were visually inspected and excluded from analysis if any voluntary muscle activity was observed in the 200 ms preceding the stimulus artefact. The peak-to-peak amplitude of MEPs and M-waves was measured in each trial, and expressed in mV. Average MEP and M-wave amplitudes were calculated for each participant at each time point (baseline, post intervention, post iTBS (when applied), 0 minutes, 10 minutes, 20 minutes and 30 minutes) for each condition (raw data are provided as S1 Dataset).

Statistical analysis was performed using SPSS Statistics (Version 18.0; SPSS, Chicago, IL). Data were tested for normality using the Kolmogorov-Smirnov statistic and transformed appropriately, if required, before analysis. Linear mixed models analysis with repeated measures were used to investigate the effects of the interventions on MEP, M-wave amplitude and SICI across each time point for the three experimental conditions. The same statistical approach was used to evaluate the effects of exercise on LTP-like plasticity by comparing iTBS under resting conditions with iTBS during low intensity exercise. Condition and time were treated as fixed effects, and each subject was considered as a random effect. Metabolic equivalents (METs) per week, obtained from the IPAQ, were considered as a covariate. Further analyses measured changes in corticomotoneuronal excitability with aerobic exercise alone by analysing the effect of time on amplitude of MEP and M-wave in the low intensity exercise condition (no iTBS), and the neuroplastic-like response in people following a stroke by analysing the amplitude of MEP and M-waves over time in the rest with iTBS condition. The significance level was set at *P* < 0.05 for all comparisons, and data are shown as mean ± standard deviation (SD) unless otherwise stated.

## Results

The interventions were well tolerated in general, but there was one drop in the first session due to experiencing dizziness from a viral infection, which was unrelated to the study protocol and these data were not included in the analysis. Some participants reported muscle soreness the day following the exercise interventions, however all recovered quickly and were able to continue participating. See Table 1 for participant characteristics.

**Table 1:** Baseline characteristics of participants (n = 12)

Age (years)	65.3 ± 7.8
Sex (F/M)	4/8
Location of stroke (L/R)	6/6
Time since stroke (months)	28 ± 41.8
Physical activity (IPAQ), MET-min/week	2,225 ± 3,192
Average heart rate during exercise conditions, beats/min	104.3 ± 13.6
Rating of Perceived Exertion during exercise conditions (Borg Scale)	12.3 ± 2.4

Values are means ± standard deviations (SD); n, number; F, female; M, male; L, left; R, right; IPAQ, International Physical Activity Questionnaire; MET, metabolic equivalents.

### M-waves

Mixed models analysis of maximal M wave amplitude revealed a significant effect of condition (F_2,177_ = 13.94, *P* < 0.001) but no effect of time (F_5,177_ = 0.398, *P* = 0.850) or time*condition (F_9,177_ = 0.370, *P* = 0.948). This was due to significant differences between Mwave amplitudes during the three conditions (Rest + iTBS mean Mmax amplitude ± SD 15.7 ± 4.9, Ex only 13.1 ± 4.6, Ex +iTBS 11.9 ± 26.2). MEP amplitudes were therefore normalised to Mmax amplitude for the remainder of the analyses.

### Motor evoked potentials

**Fig 2:**
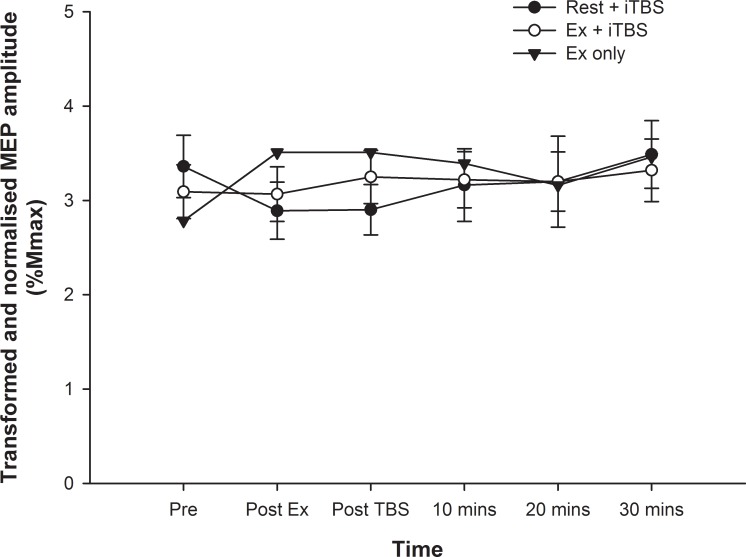
MEP amplitudes. The MEP amplitude for all three conditions are shown, normalised to Mmax for each individual and square root transformed. There was no significant main effect for time or condition, and no time*condition interaction (*P* > 0.05 for all).

After normalising MEP amplitude to Mmax for each individual (nMEP), data were square root transformed to achieve a normal distribution. Mixed models analyses revealed no significant effect of time (F_5,177_ = 0.502, *P* = 0.774), condition (F_2,177_ = 1.187, *P* = 0.307), or time*condition interaction (F_9,177_ = 0.584, *P* = 0.810) on nMEP amplitude (see Fig 2). These analyses were repeated with METs/week as a covariate, with no change in the results.

### SICI

SICI data were skewed, and square-root transformation was performed. Mixed models analysis showed there was no significant effect of time (F_5,176_ = 0.139, *P* = 0.983), condition (F_2,176_ = 1.930, *P* = 0.148), or condition*time interaction (F_9,176_ = 0.422, *P* = 0.922) on SICI2. Similarly, there was no significant difference in SICI3 across time (F_5,176_ = 0.148, *P* = 0.980), condition (F_2,176_ = 0.630, *P* = 0.534), nor a condition*time interaction (F_9,176_ = 0.045, *P* = 1.000).

### Effect of exercise on neuroplasticity

**Fig 3:**
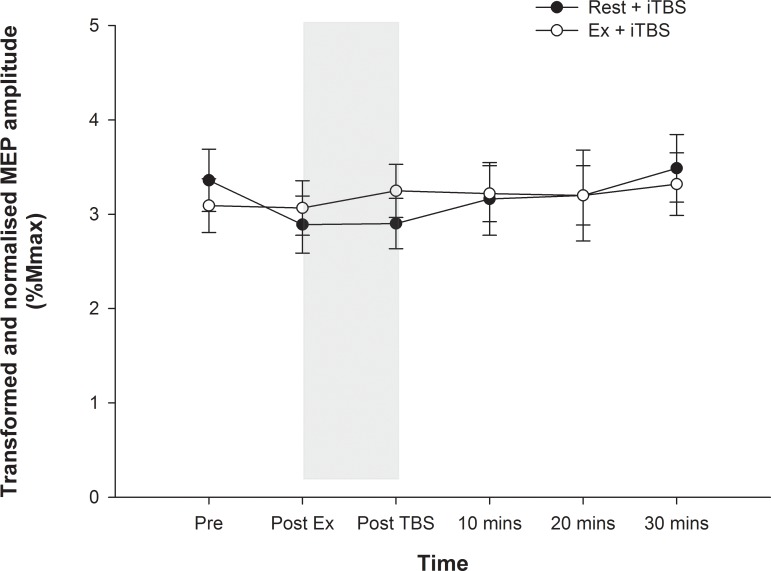
MEP amplitudes for both iTBS sessions. The MEP amplitudes, normalised to Mmax for each individual and square root transformed, are shown for the rest+iTBS and exercise+iTBS conditions. The shaded area indicates when the iTBS was delivered. There was no difference between the conditions, no change in nMEP over time, and no time*condition interaction (all *P* > 0.05).

In order to directly compare the effect of exercise on LTP-like plasticity induced by iTBS, we compared the effects of Ex+iTBS and Ex alone on nMEP amplitude. There were no significant effects of time, condition, or time*condition when comparing these two conditions (all *P* > 0.05). These results indicate that the iTBS intervention alone did not result in a significant increase in nMEP amplitude. Closer inspection of these data revealed that there was a tendency for nMEP amplitudes to decrease immediately following the rest period, with only a gradual increase in MEP amplitude following the iTBS back to the pre rest value (see Fig 3).

### Effect of exercise alone on corticomotoneuronal excitability

**Fig 4:**
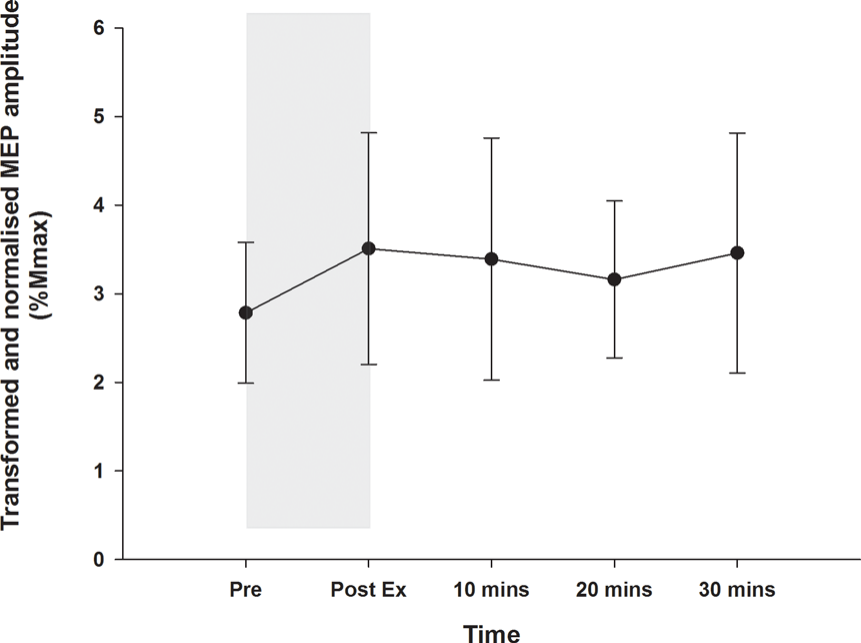
Corticomotoneuronal excitability following exercise. The MEP amplitudes, normalised to Mmax for each individual and square root transformed, are shown for the exercise only condition, with the shaded area indicating the period when 30 mins of low intensity cycling was performed. There was no change in excitability over time (*P* = 0.661).

The exercise alone condition was also examined individually to determine whether lower limb cycling influenced the corticomotoneuronal excitability in the hand. Mixed models analysis of normalised and transformed nMEP data were examined with time as a fixed effect. There was no significant change in MEP amplitude over time with exercise alone (F_4,51_ = 0.605, *P* = 0.661), see Fig 4.

## Discussion

The present study indicates that 30 minutes of low intensity cycling did not enhance LTP-like neuroplasticity in the motor cortex in individuals who were at least 6 months post-stroke. In these individuals post-stroke, iTBS alone did not induce an LTP-like response. Further, aerobic exercise had no significant effect on corticomotoneuronal excitability.

These results do not support our work in healthy young adults, which showed that 30 minutes of low intensity cycling promoted stimulation-induced plasticity [18]. There are three key differences between the studies which may explain these results. Firstly, we used a different stimulation paradigm in the present study. Rather than using continuous (inhibitory) TBS, we chose to use (facilitatory) iTBS in this study due to the robust facilitatory effects in the acute [46] and chronic stage post stroke [47]. While there has been some concern regarding the response variability with iTBS [48] since the first published report by Huang [23], recent research has supported the use of iTBS as a reliable technique to increase motor cortical excitability in young adults [49]. We attempted to control for several factors which may influence response variability (e.g. time of day, caffeine use, history of synaptic activity) but still found that only six of the 12 participants responded with an increase in excitability after iTBS, and for many participants this was only transient. Thus the difference in TBS paradigm may account for some of our findings, that exercise did not enhance stimulation-induced neuroplasticity.

A second key difference between the two studies is the age of participants. Previous studies investigating the reproducibility of TBS paradigms generally use healthy young adults, with average ages of 26.7 ± 8.1 [18] and 25.3 ± 8.7 [49]. It is well established that with ageing the ability for neuroplastic change tends to decline [50, 51]. Given the median age of our participants was 65.3 ± 7.8 years, it is likely that the failure to respond to the iTBS paradigm alone is due in part to the effect of advanced age. This highlights the need to establish the effectiveness of stimulation paradigms to induce neuroplasticity in older participants, before they can be reliably applied to the older stroke population.

Finally, the participants in our study had suffered a stroke on average two years prior to their involvement in the study. While imaging could not be obtained from all participants, as they were recruited from the community. All participants were able to perform a MVC with their affected index finger, suggesting that they had a mild middle cerebral artery stroke which left them with minimal residual hand weakness. While several studies that have investigated the effect of iTBS applied to the lesioned hemisphere in chronic stroke [47] [26], most have focused on clinical outcomes such as spasticity [52] or arm function [25] and very few have found an increase in corticomotoneuronal excitability [47]. Indeed, a Cochrane review [53] investigating repetitive stimulation paradigms to either the affected or unaffected hemispheres concluded that there is insufficient evidence that repetitive TMS improves function following stroke. Further research is needed to tailor the site and type of stimulation to individual stroke survivors, and our results suggest that further research needs to consider the impact of age on the ability to induce plasticity.

Our results did not support an exercise induced decrease in M-wave amplitude, likely as a result of an increase in body temperature after exercise [18, 41, 54]. Instead we found a significant effect of condition, with M-waves in the rest with iTBS condition being higher overall. This finding may be a result of a decrease in amplitude of M-waves in the conditions involving low intensity cycling, however no significant interaction was found between time and treatment.

Although we found no neuroplasticity response to iTBS, there was a trend towards an increase in nMEP size at 10 minutes post iTBS and 30 minutes post iTBS. Further analysis of the grouped data found there was a 12% increase in nMEP at 30 minutes post iTBS. While initial calculations suggested we needed a 25% increase in MEP size to detect a significant effect, this was powered at 80%, and currently we only have 47% power for detecting change. Post-hoc power calculations indicate that we would have needed 259 participants to detect a significant increase in nMEP amplitude between pre and 30 minutes post iTBS, with 80% power in the rest and iTBS condition. This small effect size from iTBS, and the lack of a trend towards increasing the MEP size after combining exercise and iTBS, suggests that this approach is not likely to be clinically meaningful.

Previous studies supporting the use of exercise to promote neuroplasticity have compared the effect of exercise in addition to brain stimulation (e.g. with paired associative stimulation [38, 55], or continuous TBS [18]). However, whether exercise alone influences corticomotoneuronal excitability had not yet been established. This may be an important consideration; if exercise alone has a facilitatory or depressive effect on corticomotoneuronal excitability then it may interfere with subsequent interventions. For example, if exercise were to have a facilitatory effect, then the LTD-like effect of cTBS may be enhanced (as in our previous work [18]) via homeostatic metaplasticity mechanisms [56]. However we were able to demonstrate in the present study with stroke survivors that no significant change in MEP amplitude was found over time in the aerobic exercise only condition.

There are several limitations to the present study. The sample size of 12 individuals was small, but as stated above just to see a change in the iTBS alone condition we would have needed a very large sample size. However, there was no such trend towards an increase in LTP-like plasticity with the exercise condition, so a larger sample size is unlikely to influence our primary outcome. We did not investigate the effects of different exercise intensities, which may have revealed a change in neuroplasticity with very light or more vigorous intensity exercise. It could be considered a limitation of the study that we did not include a functional measure. Previous studies demonstrating functional benefits of TMS paradigms have rarely found a correlation between MEP amplitude and function, so it is possible that participants may have experienced some functional benefits following iTBS that were not captured in this study.

## Conclusion

In this study combining low intensity aerobic exercise and iTBS did not induce changes in corticomotoneuronal excitability or neuroplasticity in participants in the chronic stage post-stroke. While aerobic exercise should remain an integral part of stroke rehabilitation programs to improve strength, fitness and endurance [57] and to reduce the morbidity and mortality from further strokes or other chronic conditions [58], the present study does not provide any evidence to support the use of aerobic exercise during stroke rehabilitation programs to facilitate neuroplasticity.

## Supporting Information

S1 Dataset MEP and Mwave amplitudes, and demographic data, for participants during the three experimental conditions. (XLSX)
